# Heterogeneity in Thymic Emigrants: Implications for Thymectomy and Immunosenescence

**DOI:** 10.1371/journal.pone.0049554

**Published:** 2013-02-27

**Authors:** Iren Bains, Andrew J. Yates, Robin E. Callard

**Affiliations:** 1 Immune Cell Biology, National Institute for Medical Research, Mill Hill, London, United Kingdom; 2 Department of Systems and Computational Biology, Albert Einstein College of Medicine, Bronx, New York, United States of America; 3 Immunobiology Unit, UCL Institute of Child Health, London, United Kingdom; 4 Centre for Mathematics and Physics in the Life Sciences and Experimental Biology (CoMPLEX), University College London, London, United Kingdom; 5 Department of Microbiology and Immunology, Albert Einstein College of Medicine, Bronx, New York, United States of America; University of Leeds, United Kingdom

## Abstract

The development of mature, antigen-inexperienced (naive) T cells begins in the thymus and continues after export into the periphery. Post-thymic maturation of naive T cells, in humans, coincides with the progressive loss of markers such as protein tyrosine kinase 7 (PTK7) and platelet endothelial cell adhesion molecule-1 (CD31). As a consequence, subpopulations of naive T cells can be recognised raising questions about the processes that give rise to the loss of these markers and their exact relationship to recent thymic emigrants (RTE). Here, we combine a mathematical survival analysis approach and data from healthy and thymectomised humans to understand the apparent persistence of populations of ‘veteran’ PTK7**^+^**T cells in thymectomised individuals. We show that a model of heterogeneity in rates of maturation, possibly linked to natural variation in TCR signalling thresholds or affinity for self-antigens, can explain the data. This model of maturation predicts that the average post-thymic age of PTK7**^+^**T cells will increase linearly with the age of the host suggesting that, despite the immature phenotype, PTK7**^+^**cells do not necessarily represent a population of RTE. Further, the model predicts an accelerated increase in the average post-thymic age of residual PTK7**^+^**T cells following thymectomy and may also explain in part the prematurely aged phenotype of the naive T cell pool in individuals thymectomised early in life.

## Introduction

The naive T cell pool develops and is maintained by a combination of input of cells from the thymus and the proliferation of circulating naive T cells [1], [2]. Immature recent thymic emigrants (RTE) continue to develop in the periphery [3] and are phenotypically distinct from their mature counterparts, being less responsive to antigen stimulation [4], [5] but more responsive to cytokines involved in naive T cell homeostasis such as IL-7 [6], [7]. This immature phenotype is thought to be transient, and although the stages of post-thymic maturation have been characterised phenotypically [3], the factors responsible for the conversion of RTE to mature status have yet to be identified.

**Figure 1:**
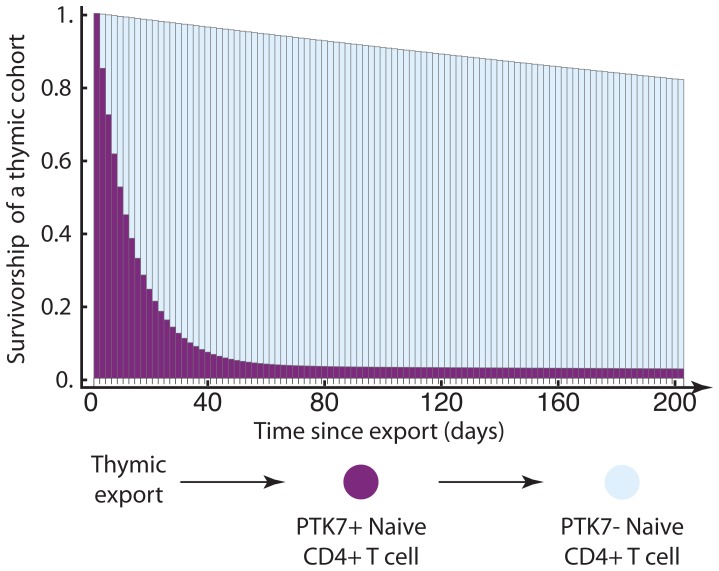
Model of post-thymic maturation of cells within the naive CD4^+^ T cell population. Survivorship of PTK7**^+^** T cells within the naive T cell pool reflects the proportion of cells that express PTK7, and are detectable in the blood, as a function of time since leaving the thymus (illustrative plot). Changes in the survivorship function might arise from maturation into PTK7^−^naive T cells, division, or death.

Studies of RTE dynamics in humans and mice have been complicated by the lack of definitive markers of RTE status. The frequency of T cell receptor excision circles (TRECs) within cell populations has been used as an indicator of time since thymic export (see, for example, ref. [8], [9]. TRECs are persistent DNA fragments that are by-products of T cell receptor (TCR) gene rearrangement during T cell development in the thymus. However, the use of TRECs to identify RTE may be inaccurate because the mean TREC content of naive T cells is influenced by both thymic export and cell division in the periphery [10]–[12]. Instead, the surface molecules CD31 (platelet endothelial cell adhesion molecule-1, PECAM-1) and PTK7 (protein tyrosine kinase 7) are used as surrogate markers of RTE status in humans [5], [13]. Circulating PTK7**^+^**and CD31**^+^**cells are enriched for TRECs, decline in frequency with age [5], [14], qualitatively in line with the normal age-related decline in thymic output, and rapidly fall in frequency following thymectomy [5], [15]. Cytokine-induced division results in the progressive loss of surface PTK7 expression *in vitro* [5] while preserving CD31 expression [6], [16]. Combined with evidence that PTK7 expression is not regained by the stimulation of PTK7^−^cells *in vitro* and PTK7**^+^**CD31^−^populations are not observed in humans [5], these observations suggest that PTK7**^+^**CD31**^+^**naive CD4**^+^**T cells are the immediate descendants of single-positive thymocytes and the precursors of more established PTK7^−^CD31**^+^**naive CD4**^+^**T cells [5] (Figure 1). Thus, the loss of this marker is thought to be a correlate of post-thymic maturation.

**Figure 2:**
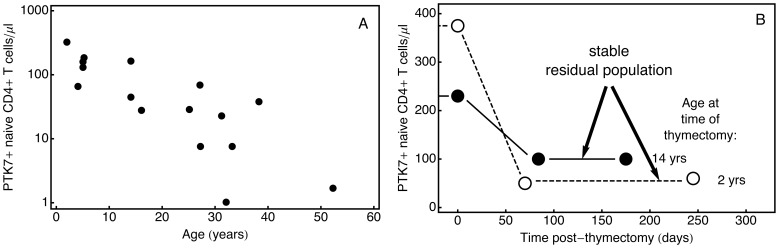
Experimental observations of PTK7^+^ T cells from Haines et al. [**5**] **.** **A:** Frequency of PTK7**^+^** naive CD4**^+^** T cells in healthy individuals aged 0 to 60 years. **B:** Frequency of PTK7**^+^** naive CD4**^+^** T cells before and after thymectomy in subjects aged 2 and 14 years.

Naive T cells from elderly individuals exhibit impaired responses to antigen [17]. This impairment is thought to arise from several sources; (i) progressive decline in the rate of export of naive T cells from the thymus, which falls approximately 20-fold from the age of 1 year to 60 years [18]; (ii) loss of TCR diversity which may itself arise through a variety of mechanisms including clonal expansion, selective recruitment into the memory population, and changes in diversity among thymic emigrants [19], [20]; and (iii) accumulated damage to long-term resident naive cells or their environment [21]. This model of age-related immunosenescence suggests that thymic function and RTE frequency in the naive population may be a useful clinical indicator of immunocompetence. However, the use of any marker such as PTK7 as a reliable indicator of RTE status relies on its expression being transient. Direct confirmation of this assumption with measurement of the duration of PTK7 expression on RTE *in vivo* has yet to be performed. Instead, our knowledge of the dynamics of its expression following thymic export derives from observations of the decline in frequency of circulating PTK7**^+^**naive CD4**^+^**T cells following thymectomy. Under these circumstances, one would expect that PTK7**^+^**populations would decline to zero, as RTE remaining after thymectomy either acquire mature status or are lost. However, Haines and colleagues [5] observed that thymectomised subjects displayed a rapid initial drop in the frequency of RTE as defined by PTK7 expression, but maintained a stable residual population of PTK7**^+^**naive CD4**^+^**T cells for at least 6 months following surgery. Further, a younger subject exhibited a substantially greater fractional loss of PTK7**^+^**cells that the older subject (Figure 2B).

The aim of the present study is to explain this persistence of PTK7 **^+^**naive CD4**^+^**T cells in thymectomised individuals with a view to understanding the mechanisms governing the transition out of the early PTK7**^+^**stage of T cell development. To do this, we develop and apply a framework that allows us to use time series measurements of the frequency of cells expressing a candidate RTE marker, following thymectomy, to characterise the dynamics of the loss of its expression within a cohort of thymic emigrants, following thymic-export (the “survivorship” of RTE, as defined by this marker).

Although the published dataset is limited, the modelling framework could still be used to reject incomplete thymectomy with residual production of T cells alone maintaining the PTK7**^+^**population as the explanation of the observations. Instead, the model predicts a long-tailed survivorship function of PTK7 residency, suggesting that naive T cells are heterogeneous with respect to their expected duration of PTK7 expression. A consequence of this heterogeneity is that despite their immature phenotype, PTK7**^+^**T cells do not necessarily represent cells that have recently left the thymus. We discuss the implications of our analysis for the age structure and functional integrity of naive T populations in elderly and thymectomised individuals.

## Methods

### The Survival Model

We use a survival model to quantify the persistence of PTK7 expression with time following thymic export. We define the *survivorship F*(*x*) as the proportion of a cohort of cells that are PTK7**^+^**and detectable *x* days post-export. Changes in the survivorship could arise from maturation to PTK7^−^status, division, or death. *In vivo* labelling studies have shown that the death of recently produced naive CD4**^+^**T cells is negligible over periods of up to 200 days [22], [23], suggesting that the loss of PTK7**^+^**naive CD4**^+^**T cells, at least during the first 6 months post-export, is almost exclusively associated with post-thymic maturation into the PTK7**^+^**naive T cell population.

Under normal physiological conditions, the total body number of cells expressing any putative RTE marker, such as PTK7, in an individual aged *t*, *X*(*t*), is determined by a convolution of thymic export and the survivorship of emigrants within the peripheral RTE marker-delineated population, as follows:
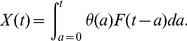
(1)


Here 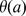 is the rate at which marker-delineated, PTK7**^+^**naive CD4**^+^**T cells, are exported by the thymus at age *a* (cells/day). *X*(*t*) is related to the number of cells per microlitre of blood by a scaling factor which accounts for blood volume and assumes a percentage of cells will be found in the lymph nodes and spleen (the exact scaling is not important since later estimates of survivorship rely on the relative change in *X*(*t*) post thymectomy). We assume that division within the PTK7^+^ subpopulation is negligible. This is justified by the observed loss of PTK7 expression on naive cells following *in vitro* cytokine-induced division [5]. However, a model that includes division within the PTK7^+^ population suggests that our results are not sensitive to this assumption (Appendix S1).

### Modelling Thymectomy

Removal of the thymus prevents further production of new PTK7^+^ cells. Assuming the survivorship function is unchanged by thymectomy (that is, there is no dependence of maturation rates on total RTE numbers), the size of the PTK7^+^ naive CD4^+^ T cell population, 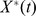, at age *t* following thymectomy at age 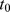, can be described as follows:
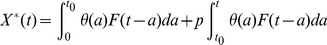
(2)


Here normal thymic production occurs up to age 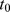, and *p* is the fraction of thymic production that continues post-thymectomy (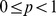).

The survivorship can be calculated from clinical observations of PTK7^+^ cell numbers, post-thymectomy. Equation (2) can be rewritten to compare the number of PTK7^+^ T cells in a healthy individual aged *t*, 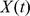, to the expected number of cells in an individual that undergoes thymectomy at age 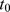, 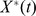, where 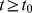. This is simply the size of the population in a non-thymectomised, age-matched individual less the cells that would have been produced between ages 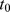 and *t*:
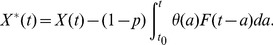
(3)


In Appendix S2, we show that in general the survivorship function, 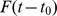 (the proportion of PTK7^+^ cells exported immediately prior to thymectomy at age 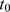 that are expected to remain at age 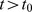) can be estimated from a (sufficiently detailed) timecourse 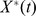 using.
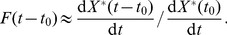
(4)


### Calculating the Post-thymic Age Distribution of PTK7^+^ Cells

Combining estimates of PTK7^+^ survivorship and thymic export, we can estimate the fraction of cells of post-thymic age 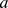 in an individual aged 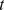, 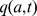:
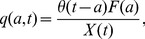
(5)where 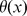 is the rate at which PTK7^+^ naive CD4^+^ T cells are exported by the thymus (cells/day) at age *x*, as before.

## Results

### Residual Thymic Output is not Sufficient to Explain the Persistent PTK7^+^ Naive CD4^+^T Cell Population Post-thymectomy

**Figure 3:**
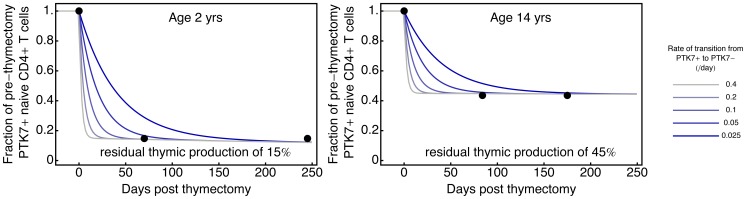
Post-thymectomy dynamics of PTK7^+^ naive CD4^+^ T cells in a model where residual thymic production alone maintains cell numbers. Residual thymic production of ∼15% and ∼45% is required to reproduce the observed persistence of PTK7**^+^** T cells in subjects thymectomised at age 2 and 14 years, respectively. Each curve corresponds to a constant rate of maturation from PTK7**^+^** to PTK7^−^ naive T cells, as described in legend. Filled circles: observations by Haines & colleagues [5].

A potential explanation for the residual stable population of PTK7^+^ cells (Figure 2B) is that thymectomy is incomplete or that extra-thymic development of T cells contributes RTE in the absence of the thymus. We demonstrate that residual thymic output alone, in the absence of variation in PTK7 maturation rates, is not sufficient to explain the observed data. The contribution of variation in rates of PTK7 maturation is excluded by assuming that PTK7 cells mature at a constant *per cell* rate 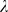 such that the survivorship function becomes a simple exponential decay (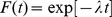). The frequency of PTK7^+^ T cells post-thymectomy in equation 2 can then be re-written as:
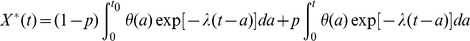


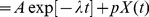
(6)

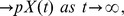
where 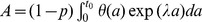 and is constant with respect to *t*. We use this model to simulate the change in PTK7^+^ naive CD4^+^ T cells post-thymectomy for varying fractions of residual thymic production, *p*, and varying rates of PTK7^+^ to PTK7^−^ transition, 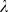. We see that residual thymic output of the order of ∼15% and ∼45% is required to explain the thymectomy data in subjects aged 2 and 14 years, respectively (Figure 3). Alternative survivorship functions (decaying slower than exponentially) can explain the data with lower residual thymic output; however, these functions implicitly assume variation in the rate of PTK7 maturation and are discussed later in the manuscript.

The experimental observations by Haines and colleagues [5] describe the progress of subjects with myasthenia gravis following thymectomy that is described as clinically complete. Typically, short-term completion studies in patients who have undergone complete thymectomy for treatment of myasthenia gravis reveal <5% residual thymic tissue [24]. Furthermore, studies of the size and thymocyte content of secondary thymic tissue in mice suggest that the contribution of ectopic thymi as an alternative source of PTK7**^+^** T cells is at least 100-fold smaller than that of the primary thoracic thymus [25]. These observations suggest that residual thymic production is likely less than approximately 5% of normal, and substantially lower than the ∼15–45% required to explain the experimental observations.We conclude that variation in PTK7 maturation rates must play a role in explaining the residual PTK7 population.

### Variation in Maturation Rates can Explain the Observations

Maintenance of a residual PTK7**^+^** population is unlikely to occur through proliferative renewal because cell division has been shown to induce loss of PTK7 expression [5]. Thus, the *per cell* maturation rate across the total peripheral PTK7**^+^** population must decline following thymectomy. This could occur through (i) heterogeneity in the maturation potential within the PTK7**^+^** population, leading to variation in the effective maturation rate with time since export (independent of thymectomy), or (ii) a homogeneous decline in the maturation rate with time since thymectomy.

Heterogeneity in the potential for maturation within the PTK7**^+^** population might arise from stochastic expression of receptors for maturation signals or levels of intracellular signalling components. Alternatively, if TCR signals are implicated in maturation, natural intra-cellular variation in avidity for self may result in a distribution of expected times to exit from the PTK7**^+^** population.

A potential biological mechanism underlying a homogeneous decline in the *per cell* rate of maturation might be a thymus-derived maturation signal (such as a cytokine or a hormone); thymectomy abrogates this signal and reduces maturation rates for all existing PTK7**^+^** cells. Alternatively, homeostatic survival signals may act to preserve a small immature naive T cell population, post-thymectomy.

#### Heterogeneous model

The heterogeneous model allows cells within each cohort of thymic emigrants to have varied potential to acquire PTK7^−^ status. To explain a persistent PTK7**^+^** population, the heterogeneous model requires that the average *per cell* maturation rate 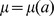 must decline with time since since export 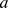. This rate is related to the survivorship through
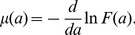
(7)


**Figure 4:**
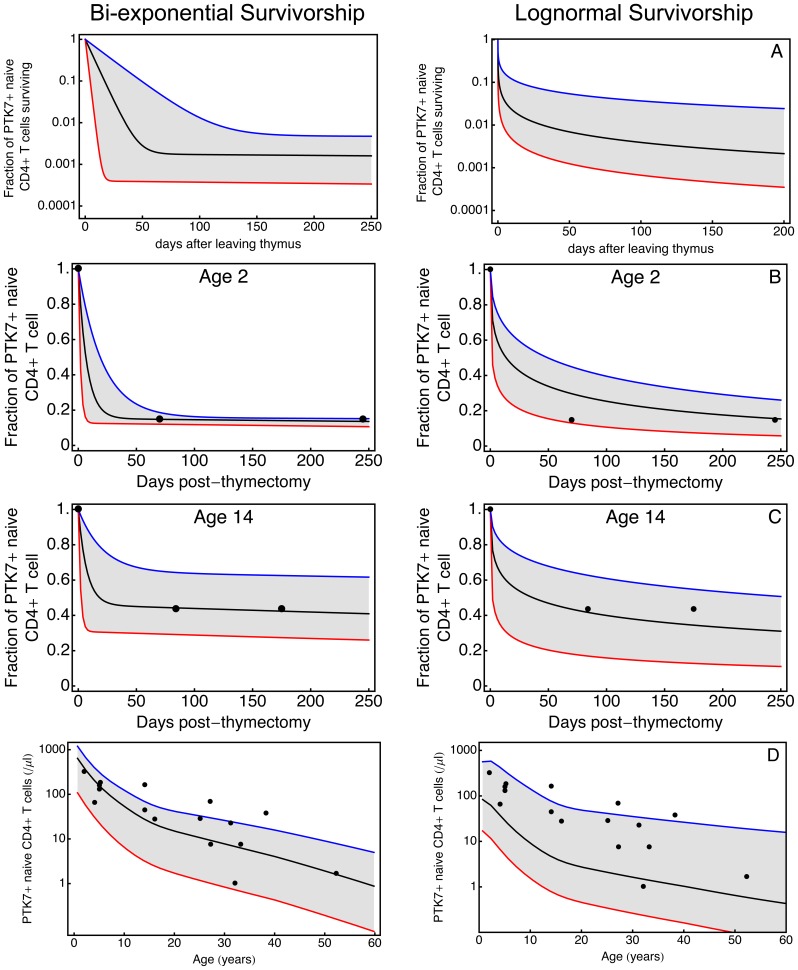
Survivorship of circulating PTK7^+^ T cells estimated by bi-exponential (left column) and lognormal distributions (right column). (**A**) PTK7 survivorship functions. Black lines: estimated survivorship using best-fit parameters. Grey regions: a family of feasible survivorship functions that encompass observations in both healthy and thymectomised individuals. (**B–C**) Decline in PTK7**^+^** naive CD4**^+^** T cells post-thymectomy predicted by survivorship functions and clinical observations following thymectomy at age 2 and 14 years (filled circles). (**D**) PTK7**^+^** naive CD4**^+^** T cell numbers in healthy individuals from birth to age 60 years simulated using feasible survivorship functions and independent estimates of thymic export [26]. Experimental observations (filled circles) are as published by Haines et al. [5].

Thus any distribution of survival times that generates 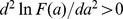 is a candidate. Examples are the bi-exponential (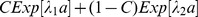) and log-normal distributions. We used the data from thymectomised patients to estimate the parameters of these distributions (Figure 4A). A Nelder-Mead optimisation is used to identify a single set of parameters that simultaneously describe the fractional decline in PTK7**^+^** T cell numbers following thymectomy in both patients. The limited data obviously provides weak constraints on these parameters. Nevertheless, as a consistency check, we used the point estimates of the survivorship parameters to simulate the numbers of PTK7**^+^** cells with age in healthy individuals. This requires an estimate of thymic output as a function of age, obtained previously [26]. Convolutions of the predicted survivorships and thymic output (equation 1) are able to reasonably describe the PTK7**^+^** naive CD4**^+^** T cells dynamics in both thymectomised and healthy individuals (Figure 4, rows A and C). The bi-exponential model appears to give a better fit to the data.

#### Homogeneous models

Numbers of PTK7**^+^** naive CD4**^+^** T cells post-thymectomy can also be modelled by a homogeneous time-dependent maturation rate 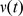. In this model, all cells have an equal probability of maturing within any time interval. This yields
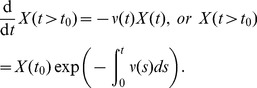
(8)


The trend in the data suggests that 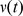 must decline with time following thymectomy, but there is flexibility in its parameterisation. A reasonable form is a sigmoidal function which saturates at a PTK7 density 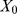,
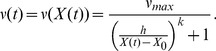
(9)


**Figure 5:**
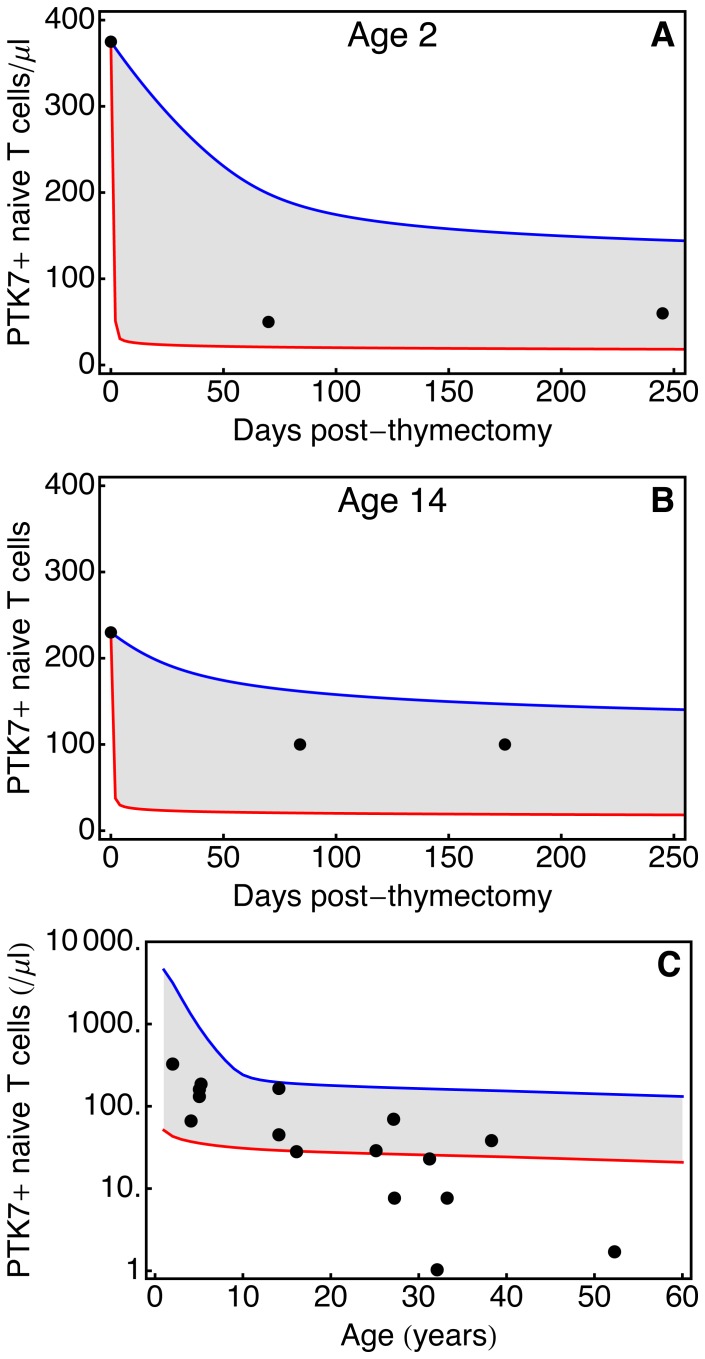
Homogeneous rate of PTK7+ T cell maturation. (**A–B**) Decline in PTK7**^+^** naive CD4**^+^** T cells post-thymectomy predicted by a range of density-dependent functions and clinical observations made by Haines et al. [5]. (**C**) Age-related change in PTK7+ T cells with age predicted by the same family of density-dependent maturation functions combined with independent estimates of thymic export [26]. Filled circles are experimental observations from Haines et al. [5]. Grey region: a family of functions defined by equation (9) (where 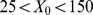) that encompasses observation in thymectomised individuals aged 2 and 14 years.

A sigmoidal function describes PTK7**^+^** numbers in the thymectomised patients reasonably for choices of 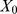 in the range 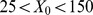 cells per 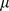
l (Figure 5 A–B; grey region). However, the same density dependent function fail to give a good fit to data from healthy individuals (Figure 5C). (An alternative, strictly time-dependent function would describe the data from thymectomised patients equally well, but provides no information regarding the behaviour of PTK7 in healthy individuals with age.).

### Discriminating between Models of Variation in RTE Maturation Rates

#### The RTE heterogeneity model predicts a broader age distribution of PTK7**^+^** cells than the homogeneous model

**Figure 6:**
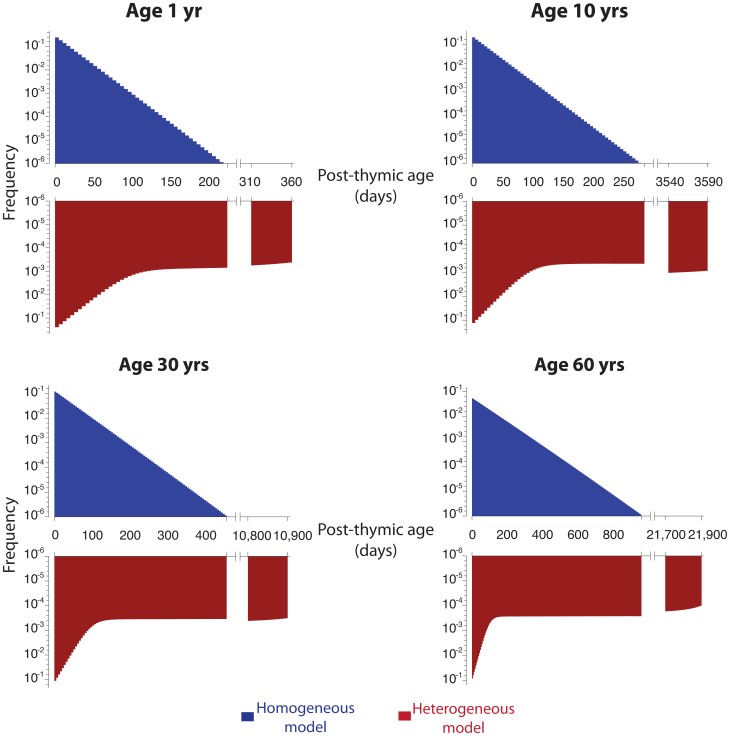
PTK7^+^ dynamics in a healthy individual. Post-thymic age distribution of PTK7**^+^** naive CD4**^+^** T cells, in typical 1, 10, 30 and 60 year olds, calculated using the homogeneous (**blue**) and heterogeneous (**red**) models. The homogeneous model predicts an exponential distribution of post-thymic age (mean post-thymic age ∼0.25 years in a 60 year old subject); the heterogeneous model predicts an increasingly broad post-thymic age distribution (and significant accumulation of veteran PTK7**^+^** cells) in aged individuals (mean post-thymic age ∼15 years in a 60 year old subject).

Both classes of model predict the post-thymic age distribution of PTK7**^+^** cells, 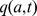 (Equation 5). This distribution is approximately exponential in the homogeneous model (Figure 6), because the timescale of changes in thymic output in healthy people (which falls approximately 20-fold between age 1 and age 60; ref. [18] is much longer than the expected residence time of cells in the PTK7**^+^** compartment. A mean per-cell rate of PTK7 loss (in healthy individuals) can be estimated by combining estimates of thymic output with age [26] and the observed PTK7**^+^** numbers with age (Figure 2A); using this to parameterise the homogeneous model predicts that the mean residence time of cells in the PTK7**^+^** compartment increases from roughly 17 days at age 1 to 90 days at age 60 (Figure 6).

The heterogeneous model predicts a decline in the frequency of most-recently emigrated PTK7**^+^** T cells, and accumulation of veteran PTK7**^+^** T cells, in the elderly (Figure 6). Using the best-fit parameters for the bi-exponential survivorship (Figure 4, left-hand column) the mean post-thymic age increases from approximately 25 days at age 1 to approximately 15 years at age 60.

#### The RTE heterogeneous model predicts a steeper increase in the mean post-thymic age of PTK7**^+^** T cells following thymectomy than the homogeneous model

**Figure 7:**
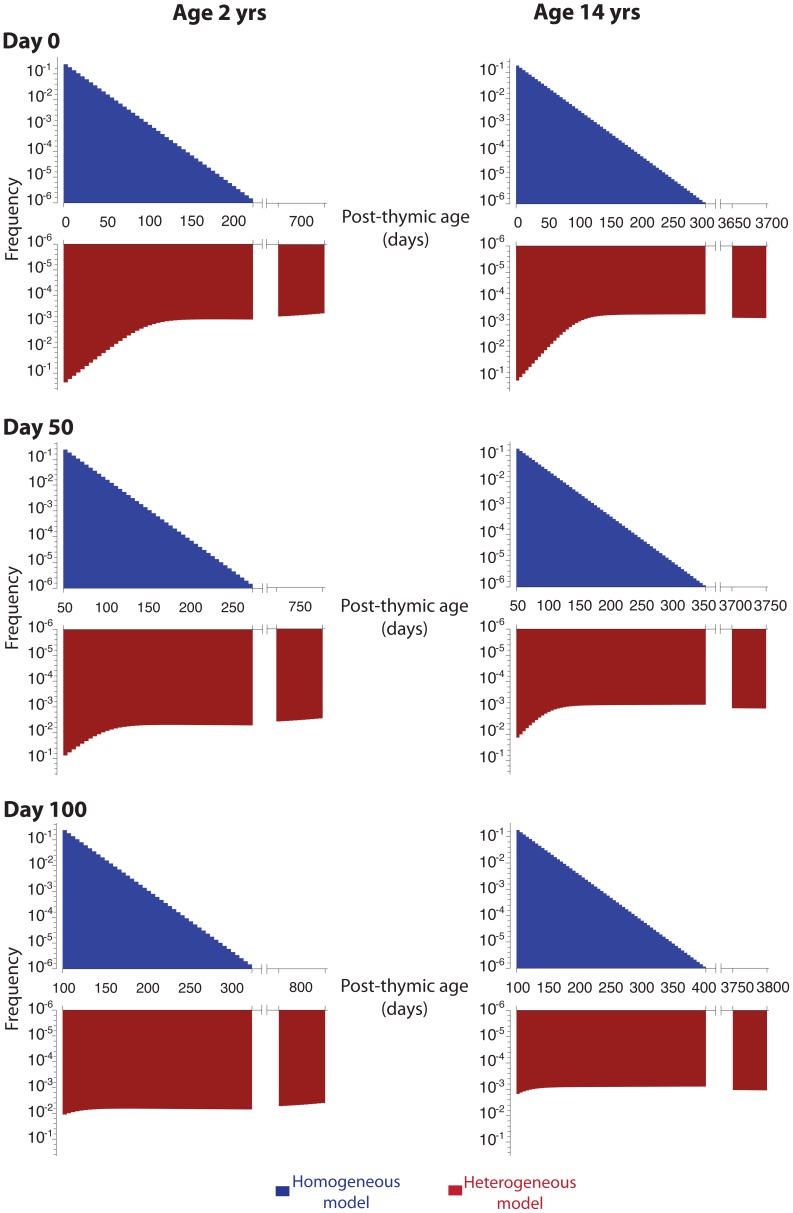
Implications of thymectomy. Post-thymic age distribution of PTK7**^+^** naive CD4**^+^** T cells at days 0, 50 and 100 following thymectomy in a 2 and 14 year old, calculated using the homogeneous (**blue**) and heterogeneous (**red**) models.

In the homogeneous model, PTK7**^+^** naive CD4**^+^** T cells mature at a rate independent of their post-thymic age. Thus whatever the dependence of the maturation rate 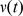 on time post-thymectomy, the distribution of PTK7**^+^** naive CD4**^+^** T cells residence times remains exponential, with mean increasing in direct proportion to time (Figure 7). In contrast, in the heterogeneous model, the PTK7**^+^** population at any instant comprises veteran, persistent cells and a rapidly maturing subpopulation that has recently been exported from the thymus. Following thymectomy the latter population disappears rapidly, leaving the veteran population and shifting the age distribution upwards (Figure 7). Thus the mean age of PTK7**^+^** naive CD4**^+^** T cells post-thymectomy increases rapidly at first, followed by a slower increase in direct proportion to age. In this model, then, thymectomy has the effect of accelerating the aging of the PTK7**^+^** population; the heterogeneous model predicts an average post-thymic age of 310 days (50 days post-thymectomy in a 2 year old) compared to an average age of 56 days (pre-thymectomy). The homogeneous model predicts an increase of only 50 days over the same period, from an average age of 18 days (pre-thymectomy) to 68 days (50 days post-thymectomy).

**Figure 8:**
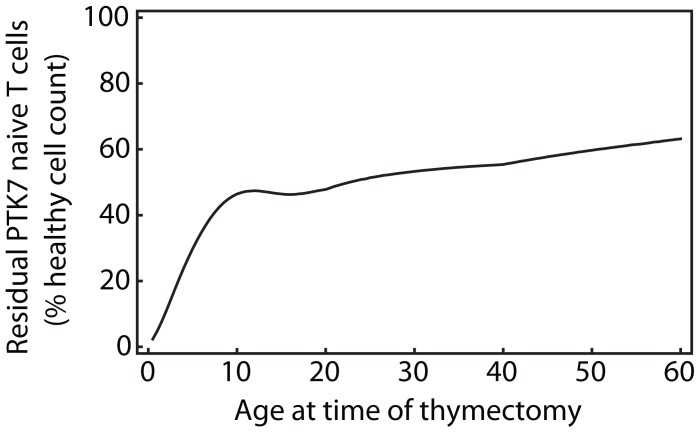
Implications of thymectomy. Predicted size of the residual PTK7**^+^** naive CD4**^+^** T cell population following thymectomy at different ages, as a percentage of expected PTK7**^+^** numbers in age-matched non-thymectomised individuals, according to the heterogeneous model (using best-fit parameters for a bi-exponential distribution guided by data from thymectomised individuals; 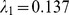, 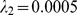, 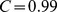).

Furthermore, the heterogeneous model naturally predicts the size of the persistent PTK7**^+^** T cell population will increase with age at time of thymectomy (Figure 8). This is a direct corollary of the predicted shift in an aging host towards a PTK7**^+^** compartment that is increasingly dominated by veteran, persistent cells.

## Discussion

Thymic emigrants play a central role in generating and maintaining the diversity of our T cell repertoires. They represent a phenotypically distinct subset of naive T cells; RTE survive preferentially over quiescent, mature naive cells [23], [27]. However the rules governing post-thymic maturation have yet to be identified. Here a novel application of survival analysis, in combination with published data from thymectomised subjects, is used to explore the incorporation of cells into the mature naive T cell pool, in which immature cells are identified by PTK7 expression. It is shown that residual thymic output alone, in the absence of variation in maturation rates, is not sufficient to explain the observed decay of PTK7**^+^** cells. Two models of PTK7 loss are investigated, in which the effective first-order maturation rate varies with: (i) time since export (independent of thymectomy); or (ii) time since thymectomy. The first model proposes that changes in effective rates of maturation arise from heterogeneity in maturation potential within each cohort of thymic emigrants, while the second assumes a homogeneous cohort of emigrants in which maturation rates vary across the entire population, perhaps due to homeostatic mechanisms, or loss of environmental signals derived from thymic tissue.

There is currently no experimental evidence to support a thymus-derived maturation signal that might cause a homogeneous decline in the rate of maturation post-thymectomy. Further, thymectomy is associated with increased naive T cell proliferation [28] which we might expect to result in increased PTK7**^+^** T cell maturation, since division is associated with loss of PTK7 expression.

We argue that the heterogeneous model provides the simplest explanation for the observed dynamics post-thymectomy; that the residual population represents a subpopulation of naturally persistent PTK7**^+^** naive T cells. Further, the heterogeneous model makes some clear predictions: (i) the average post-thymic age of PTK7**^+^** T cells will rapidly increase in response to both natural aging and thymectomy; and (ii) the size of the residual post-thymectomy PTK7**^+^** population will increase with age at time of thymectomy.

### RTE Heterogeneity and Age-related Immune Impairment

Naive T cells from aged individuals are impaired in their ability to proliferate and secrete cytokines in response to antigen [17] and to distinguish between self and non-self [29]. Aging is also associated with increased susceptibility to infection and diminished vaccine efficacy [30], [31]. Our understanding of the mechanisms underlying these age-associated changes is incomplete. Studies in mice have suggested that naive CD4**^+^** T cells accumulate functional defects with time spent in circulation [32]–[34] and that changes in the aged environment and atrophy of T cell precursors might lead to age-associated changes in T cells [21], [35]. Heterogeneity naturally leads to the preferential accumulation of persistent cells with age, as a result, the residual PTK7**^+^** T cell population in the elderly, which makes up approximately 10% of the naive cells, are not recent thymic emigrants but are exceptionally long-lived cells. As well as acquiring functional defects with time, these cells may be intrinsically less responsive to antigen, reflected in their failure to mature. The accumulation of ‘veteran RTE’ within this subpopulation may make an additional contribution to the observed decline in naive cell function with age.

### Thymectomy Mirrors Age-related Immune Impairment

Thymectomy early in life may lead to premature immunosenescence [15], [28], [36], [37]. For example, thymectomised children show a delayed response to tick-borne encephalitis virus vaccine that is more typical of elderly subjects [38]. The heterogeneity model predicts that the residual PTK**^+^** population in thymectomised individuals is highly enriched for veteran PTK7**^+^** T cells, as observed in older individuals. We propose that this rapid shift in the post-thymic age distribution contributes to the aged phenotype of thymectomised individuals. The accumulation of veteran cells also provides a natural explanation for the persistence of a larger residual population observed in subjects thymectomised at a later age, despite smaller initial RTE numbers in these older individuals.

### Potential Sources of Variation in the Potential of RTE to Mature

RTE undergo continued development in the periphery [3]. The homogeneous model implies that all RTE have the potential to complete this developmental process. In contrast, the heterogeneous model assumes cell-cell variation in RTE maturation potential. For example, there may be a temporal window of opportunity for acquisition of mature status, deriving perhaps from declining expression of co-stimulatory or signaling molecules from time of export; the baseline levels of these factors may vary from cell to cell. Another possibility is that variation derives from heterogeneity in responsiveness to maturation stimuli – for example, natural variation in TCR avidity for self antigens, expression of regulators of TCR signalling such as CD5 [39], Il-7 receptor expression [40] or intracellular signalling proteins [41], [42]. It is known that homeostatic proliferative potential varies considerably among T cells expressing different TCRs [43]. A corollary to the TCR signalling model is that the recruitment of RTE into the naive T cell population involves an element of post-thymic positive selection. The PTK7**^+^** T cell pool contains all T cells that have survived the thymic selection process; however, if RTE maturation is linked to an interaction with self-peptide-MHC complexes in the periphery, then T cells with lower signalling thresholds may differentiate into the PTK7^−^ resident naive T cell pool more rapidly than less responsive cells. This suggests an explanation for the observed disparity in antigen responsiveness between RTE and mature populations; the latter are enriched for cells with lower signalling thresholds that have passed this further step of positive selection.

Finally, the TCR signalling model generates a testable prediction regarding the phenotypic composition of the RTE and naive pools. Regulatory T cells that develop in the thymus (natural regulatory T cells, or nTreg) are thought to represent a population with relatively high affinity for self that survived negative selection [44]. If nTreg mature in the periphery according to the same rules as other thymic emigrants, the TCR avidity/sensitivity model predicts that nTreg RTE will mature preferentially. It follows that the PTK7**^+^** population will be enriched for nTregs relative to PTK7**^+^** cells, and that this enrichment will increase as thymic output declines with age.

## Supporting Information

Appendix S1 **Modeling division within the PTK7^+^ naive CD4^+^ T cell population.**
(PDF)

Appendix S2 **Estimating the survivorship directly from timecourses of PTK7^+^ numbers.**
(PDF)
