# Supplementary material for: Heterogeneity in Thymic Emigrants: Implications for Thymectomy and Immunosenescence
Source: PLoS One. 2013 Feb 27;8(2):e49554. doi: 10.1371/journal.pone.0049554 (PMC3584139; doi:10.1371/journal.pone.0049554)
Supplement: Appendix S1 — Modeling division within the PTK7+ naive CD4+ T cell population. (PDF) [file pone.0049554.s001.pdf]

S1: Modeling division within the PTK7$^+$ naive CD4$^+$ T cell population

The model of PTK7$^+$ naive CD4$^+$ T cell dynamics (equation (1)) can be extended to allow for homeostatic division within the PTK7$^+$ naive CD4$^+$ T cell population. We introduce a new variable, $M(x)$, to represent the number of division events taking place within x days of a PTK7$^+$ naive CD4$^+$ T cell leaving the thymus. The predicted size of the PTK7$^+$ naive CD4$^+$ T cell population at any given time becomes a function of thymic export, the residency time of cells and the expected number of divisions:

$$X(t) = \int_0^t \theta(x) F(t-x) 2^{E[M(t-x)]} dx \tag{10}$$

where $E[M(x)]$ is the expected number of divisions occurring in x days of exit from the thymus. In the absence of experimental data, we make the parsimonious assumption that the residency time of PTK7$^+$ naive CD4$^+$ T cell is independent of homeostatic division events.

Using the same approach described in the methods, we compare equation (**??**) to a corresponding expression for the decay of PTK7$^+$ naive CD4$^+$ T cell following thymectomy, allowing for division, to derive an expression for the survivorship function of peripheral PTK7$^+$ naive CD4$^+$ T cells:

$$F\left(t-t_0\right) \approx \frac{\mathrm{d}X^*(t-t_0)}{\mathrm{d}t} / \left(\frac{\mathrm{d}X^*(t_0)}{\mathrm{d}t} 2^{q(t-t_0)} \right) \tag{11}$$

where, in the absence of experimental data we assume that the probability of a PTK7$^+$ naive CD4$^+$ T cell dividing on any given day is constant, q, and independent of time since export. Hence the expected number of divisions in $t - t_0$ days becomes $q(t - t_0)$.

Observations of PTK7$^+$ naive CD4$^+$ T cells following thymectomy alone do not allow us to disentangle the effect of cell persistence and expansion, but it can be shown that the underlying per-cell rate of maturation, $\tilde{\mu}(a)$, for a model in which PTK7$^+$ T cells undergo homeostatic division at some constant rate, q (day^{-1}), will be higher by a constant $qLog[2]$ as compared to a model with no division, $\tilde{\mu}(a) = \mu(a) + qLog[2]$

Despite the unknown rate of division, the composite function of survival and expansion of PTK7$^+$ naive CD4$^+$ T cells in equation (**??**), $F(t-x)2^{E[M(t-x)]}$, is identical to the survivorship computed in the simpler, no-division model. So although the relative contribution of expansion and survival can not be quantified, the net survivorship of cells according to their post-thymic age, or the post-thymic age of their ancestors, can still be estimated. As a result, predicted post-thymic age distributions of PTK7$^+$ naive CD4$^+$ T cells are robust to a constant rate of background division.
