# Supplementary material for: Heterogeneity in Thymic Emigrants: Implications for Thymectomy and Immunosenescence
Source: PLoS One. 2013 Feb 27;8(2):e49554. doi: 10.1371/journal.pone.0049554 (PMC3584139; doi:10.1371/journal.pone.0049554)
Supplement: Appendix S2 — Estimating the survivorship directly from timecourses of PTK7+ numbers. (PDF) [file pone.0049554.s002.pdf]

S2: Estimating the survivorship directly from timecourses of PTK7$^+$ numbers

The survivorship function can be estimated from the timecourse of PTK7$^+$ naive CD4$^+$ T cell numbers post-thymectomy, as follows:

$$\frac{\mathrm{d}X^*(t)}{\mathrm{d}t} = \frac{\mathrm{d}X(t)}{\mathrm{d}t} - \lim_{\Delta t \to 0} \frac{\int_{t_0}^{t+\Delta t}(1-p)\theta(a)F_a(t+\Delta t-a)da - \int_{t_0}^{t}(1-p)\theta(a)F_a(t-a)da}{\Delta t} \quad (12)$$

where $F_a(t)$ is the fraction of cells from a cohort of emigrants leaving the thymus at age a that remain in the PTK7$^+$ pool for t days. We let $dX(t)/dt \approx 0$ since the observed change in PTK7$^+$ naive CD4$^+$ T cell numbers is negligible in non-thymectomised children over a 6 month period (5). A change of variables ($a = a' + \Delta t$) is used to rewrite the first integral, as follows:

$$\frac{\mathrm{d}X^*(t)}{\mathrm{d}t} \approx -(1-p) \lim_{\Delta t \to 0} \frac{\int_{t_0-\Delta t}^{t}\theta(a'+\Delta t)F_{a'+\Delta t}(t-a')da' - \int_{t_0}^{t}\theta(a)F_a(t-a)da}{\Delta t}. \quad (13)$$

We make the following simplifying assumptions: (a) $\theta(a'+\Delta t) \approx \theta(a')$, where we argue that thymic production diminishes over a number of decades and hence the change over some small period Δt is likely to be neglible; and (b) $F(t-a') \approx F(t-a')$, where it is not unreasonable to assume that PTK7 survival prospects will be similar for cohorts of thymic emigrants leaving the thymus Δt days apart. As a result, equation (**??**) becomes:

$$\begin{aligned} \frac{\mathrm{d}X^*(t)}{\mathrm{d}t} &\approx -(1-p) \lim_{\Delta t \to 0} \frac{\int_{t_0-\Delta t}^{t_0}\theta(a)F(t-a)da}{\Delta t} \\ &\approx -(1-p)\theta(t_0)F(t-t_0) \end{aligned} \quad (14)$$

Since all T cells leaving the thymus are assumed to enter the PTK7$^+$ population at day 0, $F(0) = 1$ and so

$$\theta(t_0) \approx -\frac{1}{1-p}\frac{\mathrm{d}X^*(t_0)}{\mathrm{d}t}. \quad (15)$$

Substituting this into equation (**??**) gives the following expression for the survivorship function (equation (4) in the text):

$$F(t-t_0) \approx \frac{\mathrm{d}X^*(t-t_0)}{\mathrm{d}t} / \frac{\mathrm{d}X^*(t_0)}{\mathrm{d}t}. \quad (16)$$
